# 2-(4-Bromo­phen­oxy)-3-isopropyl-5,6,7,8-tetra­hydro-1-benzothieno[2,3-*d*]pyrimidin-4(3*H*)-one

**DOI:** 10.1107/S160053680803732X

**Published:** 2008-11-22

**Authors:** Hong-Mei Wang, Li-Li Chen, Ting Hu, Xiao-Hua Zeng

**Affiliations:** a Department of Medicinal Chemistry, Yunyang Medical College, Shiyan 442000, People’s Republic of China

## Abstract

In the title compound, C_19_H_19_BrN_2_O_2_S, the central thieno­pyrim­idine ring system is essentially planar, with a maximum displacement of 0.068 (3) Å. The attached cyclo­hexene ring adopts a half-chair conformation. The molecular conformation and crystal packing are stabilized by three intra­molecular C—H⋯O hydrogen bonds and two C—H⋯π inter­actions.

## Related literature

For background to the use of pyrimidine derivatives as drugs, see: Ding *et al.* (2004 ▶). For a description of the Cambridge Structural Database, see: Allen (2002 ▶). For a related structure, see: Zeng *et al.* (2006 ▶).
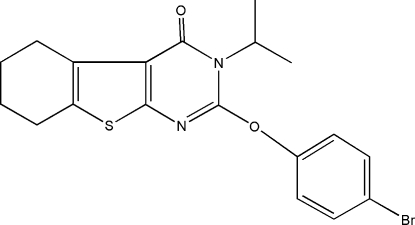


## Experimental

### 

#### Crystal data

C_19_H_19_BrN_2_O_2_S
*M* *_r_* = 418.32
Monoclinic, 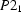

*a* = 13.3957 (7) Å
*b* = 5.7366 (3) Å
*c* = 13.3956 (7) Å
β = 115.5410 (10)°
*V* = 928.81 (8) Å^3^
*Z* = 2
Mo *K*α radiation
μ = 2.34 mm^−1^
*T* = 298 (2) K
0.20 × 0.10 × 0.10 mm

#### Data collection

Bruker SMART CCD area-detector diffractometer
Absorption correction: multi-scan (*SADABS*; Sheldrick, 1996 ▶) *T*_min_ = 0.652, *T*_max_ = 0.800
5798 measured reflections
3228 independent reflections
2346 reflections with *I* > 2σ(*I*)
*R*_int_ = 0.106

#### Refinement

*R*[*F* ^2^ > 2σ(*F* ^2^)] = 0.067
*wR*(*F* ^2^) = 0.204
*S* = 1.07
3228 reflections
228 parameters
1 restraint
H-atom parameters constrained
Δρ_max_ = 7.69 e Å^−3^
Δρ_min_ = −2.63 e Å^−3^
Absolute structure: Flack (1983 ▶), 1424 Freidel pairs
Flack parameter: 0.00 (8)

### 

Data collection: *SMART* (Bruker, 2001 ▶); cell refinement: *SAINT* (Bruker, 2001 ▶); data reduction: *SAINT*; program(s) used to solve structure: *SHELXS97* (Sheldrick, 2008 ▶); program(s) used to refine structure: *SHELXL97* (Sheldrick, 2008 ▶); molecular graphics: *PLATON* (Spek, 2003 ▶); software used to prepare material for publication: *SHELXTL* (Sheldrick, 2008 ▶).

## Supplementary Material

Crystal structure: contains datablocks global, I. DOI: 10.1107/S160053680803732X/at2674sup1.cif

Structure factors: contains datablocks I. DOI: 10.1107/S160053680803732X/at2674Isup2.hkl

Additional supplementary materials:  crystallographic information; 3D view; checkCIF report

## Figures and Tables

**Table 1:** Hydrogen-bond geometry (Å, °)

*D*—H⋯*A*	*D*—H	H⋯*A*	*D*⋯*A*	*D*—H⋯*A*
C11—H11⋯O1	0.98	2.20	2.726 (10)	112
C12—H12*B*⋯O2	0.96	2.43	2.915 (13)	111
C13—H13*A*⋯O2	0.96	2.38	2.951 (10)	117
C12—H12*A*⋯*Cg*1^i^	0.96	2.92	3.854 (11)	165
C12—H12*A*⋯*Cg*2^i^	0.96	2.71	3.434 (11)	133

Symmetry code: (i) 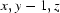. *Cg*1 and *Cg*2 are the centroids of the thiophene (S1/C5–C8) and pyrimidine (N1/N2/C7–C10) rings, respectively.

**Fig. 1.**
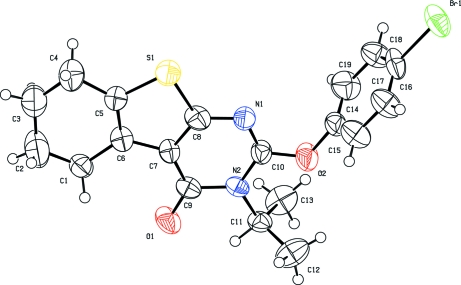
View of the molecule of (I) showing the atom-labeling scheme. Displacement ellipsoids are drawn at 50% probability level. H-atoms are represented by circles of arbitrary size.

## supplementary crystallographic information

### Comment

Pyrimidine derivatives are attracting the increasing attention of synthetic community because of the important role played by such systems in many natural products, antibiotics and drugs (Ding *et al.*, 2004). In recent years, we have been engaged in the preparation of the derivatives of heterocycles *via* aza-Wittig reaction. The title compound, (I), was synthesized and structurally characterized in this context.

The molecular structure indicates that the thieno[2,3-*d*]pyrimidine moiety is a conjugated system (Fig. 1). All ring atoms in thieno[2,3-*d*]pyrimidine are essentially coplanar (Zeng *et al.*, 2006). The bond lengths and angles are within experimental error, in the ranges of values in previously reported structures in the Cambridge Structural Database (Version 5.26; Allen, 2002).

The cyclohexene ring adopts a half-chair conformation. The crystal packing is stabilized by three intramolecular C—H···O hydrogen bonds and two C—H···π interactions (Table 1). There exist no intermolecular hydrogen bonding interactions and no π-π stackings.

### Experimental

To a solution of iminophosphorane (1.45 g, 3 mmol) in anhydrous dichloromethane (15 ml) was added iso-propyl isocyanate (3 mmol) under dry nitrogen at room temperature. After the reaction mixture was left unstirred for 48 h at room temperature, the solvent was removed off under reduced pressure and ether/petroleum ether (1:2 *v*/*v*, 20 ml) was added to precipitate triphenylphosphine oxide. After filtration, the solvent was removed, and the residue was dissolved in CH_3_CN (15 ml). After adding 4-Br-PhOH (3.1 mmol) and excess K_2_CO_3_ to the solution of carbodiimide, The mixture was stirred for 24 h at room temperature, the solution was condensed and the residue was recrystallized by EtOH to give the title compound, (I), in yield of 80% (m.p. 478 K). Elemental analysis calculated for C_19_H_19_BrN_2_O_2_S: C 54.42, H 4.57, N 6.68. Found: C 54.56, H 4.42, N 6.53. Crystals suitable for single crystal X-ray diffraction were obtained by vapor diffusion of hexane and dichloromethane (1:3 *v*/*v*) at room temperature.

### Refinement

H atoms were placed at calculated positions and treated as riding atoms, with C—H = 0.93–0.98 Å, and *U*_iso_(H) = 1.2*U*_eq_(C) for CH or 1.5*U*_eq_(C) for CH_3_.

### Crystal data

**Table:**

C_19_H_19_BrN_2_O_2_S	*F*_000_ = 428
*M**_r_* = 419.33	*D*_x_ = 1.499 Mg m^−^^3^
Monoclinic, *P*2_1_	Melting point: 478K K
Hall symbol: P 2yb	Mo *K*α radiation λ = 0.71073 Å
*a* = 13.3957 (7) Å	Cell parameters from 2048 reflections
*b* = 5.7366 (3) Å	θ = 2.9–24.5º
*c* = 13.3956 (7) Å	µ = 2.34 mm^−^^1^
β = 115.5410 (10)º	*T* = 298 (2) K
*V* = 928.81 (8) Å^3^	Block, colorless
*Z* = 2	0.20 × 0.10 × 0.10 mm

### Data collection

**Table:**

Bruker SMART CCD area-detector diffractometer	3228 independent reflections
Radiation source: fine-focus sealed tube	2346 reflections with *I* > 2σ(*I*)
Monochromator: graphite	*R*_int_ = 0.106
*T* = 298(2) K	θ_max_ = 25.0º
φ and ω scans	θ_min_ = 1.7º
Absorption correction: multi-scan(SADABS; Sheldrick, 1996)	*h* = −13→15
*T*_min_ = 0.652, *T*_max_ = 0.800	*k* = −6→6
5798 measured reflections	*l* = −15→15

### Refinement

**Table:**

Refinement on *F*^2^	Hydrogen site location: inferred from neighbouring sites
Least-squares matrix: full	H-atom parameters constrained
*R*[*F*^2^ > 2σ(*F*^2^)] = 0.067	*w* = 1/[σ^2^(*F*_o_^2^) + (0.1151*P*)^2^] where *P* = (*F*_o_^2^ + 2*F*_c_^2^)/3
*wR*(*F*^2^) = 0.204	(Δ/σ)_max_ = 0.001
*S* = 1.07	Δρ_max_ = 0.67 e Å^−^^3^
3228 reflections	Δρ_min_ = −0.62 e Å^−^^3^
228 parameters	Extinction correction: none
1 restraint	Absolute structure: Flack (1983), 1424 Friedel pairs
Primary atom site location: structure-invariant direct methods	Flack parameter: 0.00 (8)
Secondary atom site location: difference Fourier map	

### Special details

**Table:**

Geometry. All esds (except the esd in the dihedral angle between two l.s. planes) are estimated using the full covariance matrix. The cell esds are taken into account individually in the estimation of esds in distances, angles and torsion angles; correlations between esds in cell parameters are only used when they are defined by crystal symmetry. An approximate (isotropic) treatment of cell esds is used for estimating esds involving l.s. planes.
Refinement. Refinement of F^2^ against ALL reflections. The weighted R-factor wR and goodness of fit S are based on F^2^, conventional R-factors R are based on F, with F set to zero for negative F^2^. The threshold expression of F^2^ > 2sigma(F^2^) is used only for calculating R-factors(gt) etc. and is not relevant to the choice of reflections for refinement. R-factors based on F^2^ are statistically about twice as large as those based on F, and R- factors based on ALL data will be even larger.

### Fractional atomic coordinates and isotropic or equivalent isotropic displacement parameters (Å^2^)

**Table:**

	*x*	*y*	*z*	*U*_iso_*/*U*_eq_	
Br1	0.84533 (6)	0.7555 (2)	0.71718 (7)	0.0762 (4)	
C1	0.0942 (6)	1.2447 (18)	0.8813 (6)	0.0534 (18)	
H1A	0.0444	1.3157	0.8118	0.064*	
H1B	0.0650	1.0921	0.8853	0.064*	
C2	0.0967 (9)	1.390 (3)	0.9738 (10)	0.103 (5)	
H2A	0.0231	1.4514	0.9536	0.124*	
H2B	0.1152	1.2908	1.0380	0.124*	
C3	0.1719 (9)	1.578 (2)	1.0038 (11)	0.093 (4)	
H3A	0.1730	1.6464	1.0704	0.111*	
H3B	0.1416	1.6942	0.9459	0.111*	
C4	0.2917 (7)	1.537 (2)	1.0246 (7)	0.067 (3)	
H4A	0.3234	1.6797	1.0119	0.081*	
H4B	0.3351	1.4885	1.1006	0.081*	
C5	0.2930 (6)	1.3513 (13)	0.9472 (6)	0.0460 (19)	
C6	0.2071 (5)	1.2157 (15)	0.8824 (5)	0.046 (2)	
C7	0.2363 (6)	1.0548 (14)	0.8163 (6)	0.0405 (16)	
C8	0.3446 (6)	1.0816 (16)	0.8335 (6)	0.052 (2)	
C9	0.1666 (6)	0.9073 (15)	0.7312 (6)	0.0457 (18)	
C10	0.3302 (6)	0.8295 (14)	0.7015 (6)	0.047 (2)	
C11	0.1472 (6)	0.6545 (14)	0.5719 (6)	0.0459 (18)	
H11	0.0726	0.6617	0.5683	0.055*	
C12	0.1754 (9)	0.3997 (19)	0.5775 (8)	0.079 (3)	
H12A	0.1906	0.3406	0.6497	0.119*	
H12B	0.2395	0.3796	0.5638	0.119*	
H12C	0.1142	0.3161	0.5228	0.119*	
C13	0.1386 (8)	0.767 (2)	0.4657 (6)	0.071 (2)	
H13A	0.2104	0.7707	0.4661	0.107*	
H13B	0.1112	0.9233	0.4608	0.107*	
H13C	0.0889	0.6784	0.4033	0.107*	
C14	0.4794 (6)	0.7242 (18)	0.6612 (7)	0.055 (2)	
C15	0.5512 (8)	0.553 (2)	0.7233 (8)	0.073 (3)	
H15	0.5268	0.4320	0.7536	0.087*	
C16	0.6596 (8)	0.563 (2)	0.7394 (9)	0.074 (3)	
H16	0.7096	0.4498	0.7815	0.088*	
C17	0.6938 (5)	0.741 (2)	0.6934 (6)	0.061 (2)	
C18	0.6221 (7)	0.9049 (19)	0.6317 (8)	0.066 (2)	
H18	0.6458	1.0245	0.6002	0.079*	
C19	0.5134 (8)	0.895 (2)	0.6152 (9)	0.073 (3)	
H19	0.4634	1.0073	0.5720	0.088*	
N1	0.3955 (5)	0.9634 (13)	0.7793 (6)	0.0527 (17)	
N2	0.2193 (4)	0.7931 (13)	0.6717 (4)	0.0415 (13)	
O1	0.0683 (4)	0.8637 (12)	0.7061 (5)	0.0664 (19)	
O2	0.3666 (4)	0.7053 (13)	0.6384 (5)	0.072 (2)	
S1	0.41333 (15)	1.2916 (5)	0.93045 (17)	0.0604 (6)	

### Atomic displacement parameters (Å^2^)

**Table:**

	*U*^11^	*U*^22^	*U*^33^	*U*^12^	*U*^13^	*U*^23^
Br1	0.0420 (4)	0.1275 (10)	0.0622 (5)	−0.0010 (6)	0.0253 (4)	−0.0105 (6)
C1	0.048 (4)	0.055 (5)	0.061 (4)	−0.001 (5)	0.027 (3)	0.001 (5)
C2	0.063 (6)	0.162 (13)	0.089 (8)	−0.006 (7)	0.037 (6)	−0.052 (8)
C3	0.079 (7)	0.105 (10)	0.106 (9)	−0.008 (7)	0.051 (7)	−0.036 (8)
C4	0.059 (5)	0.090 (7)	0.043 (4)	0.009 (5)	0.013 (4)	−0.009 (5)
C5	0.045 (4)	0.050 (5)	0.038 (4)	0.004 (3)	0.014 (3)	0.001 (3)
C6	0.039 (4)	0.057 (6)	0.036 (3)	0.007 (4)	0.011 (3)	0.004 (4)
C7	0.032 (3)	0.052 (4)	0.033 (3)	0.007 (3)	0.010 (3)	0.002 (3)
C8	0.033 (4)	0.070 (6)	0.043 (4)	−0.004 (4)	0.006 (3)	−0.002 (4)
C9	0.037 (4)	0.055 (5)	0.040 (4)	−0.004 (4)	0.012 (3)	0.006 (3)
C10	0.038 (4)	0.053 (5)	0.049 (4)	0.004 (3)	0.018 (3)	−0.006 (3)
C11	0.037 (4)	0.053 (5)	0.044 (4)	−0.009 (3)	0.013 (3)	−0.004 (3)
C12	0.100 (8)	0.058 (6)	0.061 (6)	−0.002 (6)	0.016 (6)	0.001 (5)
C13	0.083 (6)	0.069 (6)	0.044 (4)	0.001 (6)	0.011 (4)	0.007 (5)
C14	0.039 (4)	0.068 (6)	0.061 (4)	−0.009 (4)	0.024 (3)	−0.022 (5)
C15	0.054 (5)	0.088 (8)	0.076 (7)	−0.002 (5)	0.028 (5)	0.010 (6)
C16	0.050 (5)	0.093 (8)	0.076 (6)	0.004 (5)	0.027 (5)	0.019 (6)
C17	0.034 (3)	0.106 (7)	0.043 (4)	0.005 (6)	0.016 (3)	−0.019 (6)
C18	0.049 (5)	0.072 (6)	0.074 (6)	0.000 (5)	0.023 (5)	0.016 (5)
C19	0.048 (5)	0.077 (7)	0.084 (7)	0.011 (5)	0.019 (5)	0.009 (5)
N1	0.034 (3)	0.063 (5)	0.057 (4)	−0.007 (3)	0.016 (3)	−0.022 (4)
N2	0.031 (3)	0.049 (4)	0.043 (3)	−0.005 (3)	0.013 (2)	0.002 (3)
O1	0.033 (3)	0.103 (6)	0.062 (3)	−0.012 (3)	0.018 (2)	−0.019 (3)
O2	0.039 (3)	0.098 (6)	0.079 (4)	−0.011 (3)	0.025 (3)	−0.041 (4)
S1	0.0373 (9)	0.0736 (16)	0.0600 (11)	−0.0053 (11)	0.0114 (8)	−0.0194 (12)

### Geometric parameters (Å, °)

**Table:**

Br1—C17	1.916 (7)	C10—O2	1.347 (9)
C1—C2	1.481 (14)	C10—N2	1.378 (8)
C1—C6	1.515 (9)	C11—N2	1.497 (9)
C1—H1A	0.9700	C11—C12	1.504 (14)
C1—H1B	0.9700	C11—C13	1.521 (12)
C2—C3	1.410 (18)	C11—H11	0.9800
C2—H2A	0.9700	C12—H12A	0.9600
C2—H2B	0.9700	C12—H12B	0.9600
C3—C4	1.523 (14)	C12—H12C	0.9600
C3—H3A	0.9700	C13—H13A	0.9600
C3—H3B	0.9700	C13—H13B	0.9600
C4—C5	1.492 (12)	C13—H13C	0.9600
C4—H4A	0.9700	C14—C19	1.336 (15)
C4—H4B	0.9700	C14—C15	1.377 (15)
C5—C6	1.350 (11)	C14—O2	1.412 (9)
C5—S1	1.754 (8)	C15—C16	1.374 (13)
C6—C7	1.445 (10)	C15—H15	0.9300
C7—C8	1.375 (10)	C16—C17	1.370 (15)
C7—C9	1.404 (11)	C16—H16	0.9300
C8—N1	1.370 (10)	C17—C18	1.342 (14)
C8—S1	1.719 (9)	C18—C19	1.377 (12)
C9—O1	1.236 (9)	C18—H18	0.9300
C9—N2	1.431 (10)	C19—H19	0.9300
C10—N1	1.287 (10)		
			
C2—C1—C6	113.0 (7)	N2—C11—C12	114.8 (7)
C2—C1—H1A	109.0	N2—C11—C13	111.6 (7)
C6—C1—H1A	109.0	C12—C11—C13	112.1 (8)
C2—C1—H1B	109.0	N2—C11—H11	105.8
C6—C1—H1B	109.0	C12—C11—H11	105.8
H1A—C1—H1B	107.8	C13—C11—H11	105.8
C3—C2—C1	115.1 (10)	C11—C12—H12A	109.5
C3—C2—H2A	108.5	C11—C12—H12B	109.5
C1—C2—H2A	108.5	H12A—C12—H12B	109.5
C3—C2—H2B	108.5	C11—C12—H12C	109.5
C1—C2—H2B	108.5	H12A—C12—H12C	109.5
H2A—C2—H2B	107.5	H12B—C12—H12C	109.5
C2—C3—C4	120.1 (11)	C11—C13—H13A	109.5
C2—C3—H3A	107.3	C11—C13—H13B	109.5
C4—C3—H3A	107.3	H13A—C13—H13B	109.5
C2—C3—H3B	107.3	C11—C13—H13C	109.5
C4—C3—H3B	107.3	H13A—C13—H13C	109.5
H3A—C3—H3B	106.9	H13B—C13—H13C	109.5
C5—C4—C3	108.0 (8)	C19—C14—C15	121.0 (8)
C5—C4—H4A	110.1	C19—C14—O2	120.1 (9)
C3—C4—H4A	110.1	C15—C14—O2	118.6 (9)
C5—C4—H4B	110.1	C16—C15—C14	118.6 (10)
C3—C4—H4B	110.1	C16—C15—H15	120.7
H4A—C4—H4B	108.4	C14—C15—H15	120.7
C6—C5—C4	126.6 (7)	C17—C16—C15	119.9 (10)
C6—C5—S1	112.4 (5)	C17—C16—H16	120.1
C4—C5—S1	121.0 (6)	C15—C16—H16	120.1
C5—C6—C7	112.3 (6)	C18—C17—C16	120.6 (7)
C5—C6—C1	120.9 (7)	C18—C17—Br1	119.9 (8)
C7—C6—C1	126.7 (7)	C16—C17—Br1	119.5 (8)
C8—C7—C9	119.2 (7)	C17—C18—C19	119.7 (10)
C8—C7—C6	111.8 (7)	C17—C18—H18	120.1
C9—C7—C6	128.4 (6)	C19—C18—H18	120.1
N1—C8—C7	125.8 (7)	C14—C19—C18	120.2 (9)
N1—C8—S1	121.3 (5)	C14—C19—H19	119.9
C7—C8—S1	112.8 (6)	C18—C19—H19	119.9
O1—C9—C7	126.9 (7)	C10—N1—C8	113.9 (6)
O1—C9—N2	118.7 (7)	C10—N2—C9	119.9 (6)
C7—C9—N2	114.4 (6)	C10—N2—C11	122.7 (6)
N1—C10—O2	121.4 (6)	C9—N2—C11	117.2 (5)
N1—C10—N2	126.6 (7)	C10—O2—C14	117.8 (6)
O2—C10—N2	112.0 (6)	C8—S1—C5	90.6 (4)
			
C6—C1—C2—C3	36.3 (16)	Br1—C17—C18—C19	−179.7 (8)
C1—C2—C3—C4	−50.1 (18)	C15—C14—C19—C18	−1.6 (16)
C2—C3—C4—C5	34.6 (16)	O2—C14—C19—C18	−175.9 (9)
C3—C4—C5—C6	−9.8 (13)	C17—C18—C19—C14	0.5 (16)
C3—C4—C5—S1	169.1 (8)	O2—C10—N1—C8	178.4 (7)
C4—C5—C6—C7	179.3 (8)	N2—C10—N1—C8	−1.7 (13)
S1—C5—C6—C7	0.4 (8)	C7—C8—N1—C10	4.8 (13)
C4—C5—C6—C1	1.0 (12)	S1—C8—N1—C10	−173.3 (6)
S1—C5—C6—C1	−177.9 (6)	N1—C10—N2—C9	−1.2 (12)
C2—C1—C6—C5	−13.3 (13)	O2—C10—N2—C9	178.8 (7)
C2—C1—C6—C7	168.6 (10)	N1—C10—N2—C11	173.0 (8)
C5—C6—C7—C8	−1.3 (10)	O2—C10—N2—C11	−7.0 (10)
C1—C6—C7—C8	176.9 (7)	O1—C9—N2—C10	−176.8 (7)
C5—C6—C7—C9	−172.1 (8)	C7—C9—N2—C10	1.2 (10)
C1—C6—C7—C9	6.1 (13)	O1—C9—N2—C11	8.7 (10)
C9—C7—C8—N1	−4.8 (13)	C7—C9—N2—C11	−173.3 (7)
C6—C7—C8—N1	−176.6 (8)	C12—C11—N2—C10	66.0 (10)
C9—C7—C8—S1	173.4 (6)	C13—C11—N2—C10	−63.0 (10)
C6—C7—C8—S1	1.6 (9)	C12—C11—N2—C9	−119.6 (9)
C8—C7—C9—O1	179.4 (8)	C13—C11—N2—C9	111.4 (8)
C6—C7—C9—O1	−10.4 (14)	N1—C10—O2—C14	0.8 (13)
C8—C7—C9—N2	1.5 (11)	N2—C10—O2—C14	−179.2 (7)
C6—C7—C9—N2	171.7 (7)	C19—C14—O2—C10	−86.5 (11)
C19—C14—C15—C16	1.5 (15)	C15—C14—O2—C10	99.1 (10)
O2—C14—C15—C16	175.9 (9)	N1—C8—S1—C5	177.1 (8)
C14—C15—C16—C17	−0.4 (16)	C7—C8—S1—C5	−1.2 (7)
C15—C16—C17—C18	−0.6 (16)	C6—C5—S1—C8	0.4 (6)
C15—C16—C17—Br1	179.7 (8)	C4—C5—S1—C8	−178.6 (7)
C16—C17—C18—C19	0.6 (15)		

### Hydrogen-bond geometry (Å, °)

**Table:**

*D*—H···*A*	*D*—H	H···*A*	*D*···*A*	*D*—H···*A*
C11—H11···O1	0.98	2.20	2.726 (10)	112
C12—H12B···O2	0.96	2.43	2.915 (13)	111
C13—H13A···O2	0.96	2.38	2.951 (10)	117
C12—H12B···Cg1	0.96	2.92	3.854 (11)	165
C12—H12B···Cg2	0.96	2.71	3.434 (11)	133
